# Comparison of Storage Stability and In Vitro Digestion of Rice Flour-Based Yogurt Alternatives Made with *Lactobacillus rhamnosus* Lgg to Milk-Based Yogurt

**DOI:** 10.3390/foods14071129

**Published:** 2025-03-25

**Authors:** Anita Morris, Charles Boeneke, Joan M. King

**Affiliations:** 1 School of Nutrition and Food Sciences, LSU Agriculture Center, 39 Forestry Lane, 201J Animal and Food Sciences Building, LSU, Baton Rouge, LA 70803, USA; morrisanita84@gmail.com; 2 School of Animal Sciences, LSU Agriculture Center, Baton Rouge, LA 70803, USA; cboeneke@agcenter.lsu.edu

**Keywords:** rice flour, plant-based, yogurt, probiotic

## Abstract

Production of plant-based products is still on the rise. There is a need for new plant-based dairy alternatives in the food market due to lactose intolerance, allergens to dairy and nuts and a rise in gluten-free products. Rice is a key source for these types of products because it is hypoallergenic. This study focused on the comparison storage stability and in vitro digestion of milk-based yogurt (MY) to yogurt alternatives (YA) made with four different rice flours. YAs and MY were prepared using *L. delbrueckii* and *S. thermophilus* for fermentation and *L. rhamnosus* (LGG) as a probiotic. Samples were stored refrigerated for up to 28 days and analyzed for titratable acidity, pH, color, syneresis, viscosity and bacterial counts every seven days. Probiotic survivability was tested under simulated gastric and intestinal conditions. YAs had lower syneresis than MY. There were few changes in color over time. Titratable acidity was lower in YAs (0.1 to 0.5%) than in MY (1%) while pH decreased in all samples during storage. Bacteria counts were stable throughout storage in all samples. MY had higher counts of LGG at the beginning of storage which significantly decreased during exposure to in vitro gastric conditions. Under in vitro intestinal conditions, both the white rice flour YAs and the MY retained the highest levels of LGG. This study indicated that it is possible for YAs made with rice flour to be stable overtime and with survivability of probiotic bacteria under gastric conditions.

## 1. Introduction

It has been suggested that people who grapple with health issues associated with high dietary cholesterol intake, lactose intolerance or malabsorption, and milk protein allergy consider the consumption of alternative products [1]. Also, plant-based yogurt alternatives (PBYA) are of interest for younger consumer groups, because these products are perceived as healthy, natural, and low-calorie [2]. The plant-based yogurt alternatives (PBYA) market value in the United States was $400 million in 2020, and this amount is anticipated to grow to $1.3 billion by 2027 [3]. Researchers have expended significant effort in the development and production of sustainable and healthy plant-based foods [4].

PBYA are designed to be similar to traditional yogurt regarding texture, sensory characteristics, and ability to keep lactic acid bacteria (LAB) viable during prolonged storage [1]. Production of PBYA typically results from fermentation of plant aqueous extracts, such as cereals, pseudocereals, legumes, seeds, or nuts [5]. Coconut and soy have been utilized in yogurts, with soy as being a better choice due to its protein content, but it is a concern due to allergenicity [1]. The structural integrity of PBYA is a concern during production and storage due to destabilization of plant protein structure as a result of fermentation and acidification [6]. It is also important to find milk-free plant-based products due to the lactose intolerance and milk protein allergies of some people [7,8]. PBYAs have differences in protein content, coagulation ability, coagulation temperatures, and protein hydrolysis issues that affect gelation [5]. To obtain adequate texture in commercial PBYAs, thickeners and emulsifiers including protein extracts, natural gums, starches, pectin, and guar are commonly used. Starch is of particular interest due to its swelling ability and water-holding capacity, which in turn minimize syneresis [5]. Starch can be gelatinized through heat treatment, resulting in a greater viscosity of plant-based ingredients prior to fermentation. This processing step also reduces endogenous, undesirable microbes before starting the culture inoculation and helps to arrest phase separation [9]. Starch has been shown to reduce syneresis and improve viscosity, with differences depending on the starch source. Variations could be due to differences among the starches in amylose contents, starch granule structure, and gelatinization mechanisms [9].

Rice has several advantages in its use as a yogurt-like ingredient, namely, a neutral taste, hypoallergenicity, and a good ability to create a viscous gel structure after thermal processing. While white rice is more commonly consumed, brown rice with germ is more nutritionally well-rounded, moreover containing several biofunctional compounds (gamma-oryzanol, gamma-aminobutyric acid (GABA), and ferulic acid) [10]. The Frontière rice cultivar is a high-protein rice that was developed by the Louisiana State University Agricultural Center H. Rouse Caffey Rice Research Station. The protein content for the Frontière cultivar is higher than most US cultivars [11]. The increased protein content may result in different physicochemical properties than the typical US rice cultivars when used in a YA.

Cereals can be fermented by lactic acid bacteria (LAB) to improve taste and appearance. LAB fermentation produces different flavors, aromas, and textures from the original food source [12]. LAB are gram-positive, non-spore forming, cocci- or rod-shaped, facultative anaerobes that ferment carbohydrates to produce lactic acid. *Lactobacillus*, *Leuconostoc*, *Pediococcus*, and *Streptococcus* are the genera for LAB [13]. LAB can produce exopolysaccharides (EPS), which are important in the dairy industry, because the EPS can increase the viscosity and provide stability in food products like yogurt and cheese [14]. *Lactobacillus rhamnosus* LGG^®^ is an anaerobic, rod-shaped probiotic strain [15] that can produce EPSs in plant yogurt [16]. LAB are GRAS (Generally Recognized as Safe) [14] and create an acidic environment, which makes it harder for undesirable microorganisms to survive. Commercial yogurt alternatives such as soy, coconut, almond, and hemp display a lower lactic acid content (0.43/100 g) than dairy yogurt (1.11/100 g). The degree to which LAB species can convert sugars to lactic acid varies depending on the strain of LAB used during fermentation [17].

There are a limited number of studies that have been conducted on yogurts utilizing rice ingredients. One study looked at rice germ yogurt made using *Lactiplantibacillus plantarum* fortified with lysine [18]. They obtained a low titratable acidity at 0.39% with rapid growth of the bacteria. The authors found that this yogurt stimulated the immune system in mice, and short-chain fatty acids were produced that provided intestinal health. Park and Choi [19] compared two types of lactic acid bacteria to ferment 10% skim milk with rice flour added at different levels. The authors found that bacteria increased after 2 and 6 h of fermentation. They concluded that no effects were seen on microbial growth or acidity by the addition of rice flour. Another study found that *L. bulgaricus* worked best to make rice milk-based yogurt, with a reduction in pH to 4.3, and an increase in titratable acidity, while having the highest growth rate and viable counts [20]. Salih et al. [21] produced milk-based yogurt with rice flour added up to 6%. The pH increased and the titratable acidity decreased compared to control without rice flour, while syneresis decreased as the level of rice flour increased. Two studies utilized rice milk to produce yogurt [22,23]. One of these studies added polymerized whey protein for gelation. The yogurt had a compact structure, with bacterial counts greater than 10^6^ CFU/mL after 8 weeks of storage and a water-holding capacity of 95% [23]. The second study prepared rice milk through treatment with amylase, followed by the addition of casein, soybean oil, and calcium lactate [22]. After fermentation, pectin and strawberry preserve were added. The pH of the final rice-based yogurt was 3.48, with a titratable acidity of 0.86%. The bacterial count of the rice-based yogurt was 7.6 × 10^7^ CFU/g, which was greater than the commercial dairy yogurt at 3.4 × 10^9^ CFU/g. Germinated brown rice was utilized to make yogurt-like products, and it was observed that the fermentation time was lower for flour from germinated than from ungerminated brown rice [24]. Germinated brow rice yogurts had a thinner consistency than controls, which was thought to be due to lower starch content. We utilized both white and brown flours from the new Frontière rice variety to produce YAs [25]. The white rice flour yogurt had greater viscosity than brown rice flour yogurt, as the starch level was greater in the white rice flour. The use of brown rice flour resulted in longer fermentation times needed to reach pH 4.6 but higher bacteria counts, specifically for *Lactobacillus delbrueckii* subsp. *Bulgaricus*. There were minimal differences in bacterial counts in the yogurts when comparing conventional rice flour to the Frontière samples. These YAs had probiotic counts greater than the minimum recommended after fermentation [25]. The therapeutic minimum of probiotics in products is 10^6^–10^8^ cfu/g.

The survivability of bacteria in the gastrointestinal tract depends on the strain of bacteria and the dose [26]. In vitro assays are commonly used to evaluate the viability of bacteria in simulated gastric and intestinal juices [27]. The pH in the stomach can range from 1.5 up to 6.0; however, the average range is from 2.5 to 3.5. Food normally remains in the stomach for 2 to 4 h. The pH in the intestine is usually around 8, and food remains in the intestine for 1 to 4 h. Huang et al. [28] found that soy yogurt had a lower viability of lactic acid bacteria than soy yogurt containing quinoa during storage. Pachekrepapol et al., 2021 [29] found similar results with tapioca starch added to coconut yogurt. The stability of LAB during storage was thought to be related to the level of starch added and its relationship to water-holding capacity. The storage and gastrointestinal stabilities of the probiotics in new YAs made from Frontière rice flour had not yet been tested. The aim of this study was to determine the physicochemical properties and bacteria survivability of YAs produced from Frontière rice flour compared with milk yogurt during 28 days of storage. Additionally, the viability of the probiotic bacteria was determined after the YAs and milk-based yogurt were subjected to in vitro gastric and intestinal conditions.

## 2. Materials and Methods

### 2.1. Acquisition of Rice Flour

Frontière high-protein milled brown (FBRF) and white (FWRF) high-protein rice flours were donated by Cahokia Rice (McClure, IL, USA). Commercial brown (CMBRF) and white (CMWRF) rice flour was purchased from Bob’s Red Mill (Milwaukie, OR, USA). Particle size of the rice flours was analyzed in triplicate using a laser light scattering Microtrac S3500 (Microtrac Inc., Montgomeryville, PA, USA) using the wet dispersion module. The samples were suspended in isopropanol (0.1% *w*/*v*) and vortexed in a centrifuge tube. Approximately 40 mL of sample was placed in the instrument for analysis.

### 2.2. Yogurt Alternative (YA) Preparation

YAs were prepared following the procedures of Morris et al. [25]. A 200 ppm hypochlorite solution (Chlorilizer Plus, Afco Industries Inc., Alexandria, LA, USA) was used to sanitize all equipment. YAs were manufactured from FBRF, FWRF, CMWRF, and CMBRF. TIC Pretested^®^ Pectin LM 35 Powder (0.10% *w*/*w*) in aqueous solution was utilized as a thickener and mixed with the rice flour (7.87% *w*/*w*) and water (83.13% *w*/*w*) using a Vitamix Blender (Vitamix Drink Machine Plus, Cleveland, OH, USA). The mixture was heated in a Cuisinart stainless steel 6.0 qt pot. Glucose powder (1.36% *w*/*w*) (Modernist Pantry, Kitchen Alchemy) and granulated cane sugar (7.52% *w*/*w*) (Great Value) were added to the solution. The mixture was heated with constant stirring until the temperature reached 85 °C, then placed in a water bath (85 °C) for 10 min. The mixture was stirred using a stainless immersion blender (WSB33X, Quik Stik, Waring, Saluda, NC, USA), then cooled to 42 °C using an ice bath. The rice flour mixture was inoculated with two bacterial strains (*Lactobacillus delbrueckii* subsp. *bulgaricus* and *Streptococus thermophilus*) (Danisco^®^ VEGE 033) and probiotic Nutrish^®^ LGG^®^ DA (*Lactobacillus rhamnosus*) (Chr. Hansen, Hørsholm, Denmark) at 0.01% (*w*/*w*) of each bacteria. The rice flour mixture was fermented in a Yamato IC-800 Incubator (Yamato Scientific America Inc., Santa Clara, CA, USA) (41 °C) until the pH reached approximately 4.6. The YA was then placed in an ice bath to decrease the temperature to 4 °C, then placed into 6-ounce sterilized glass jars (~145 g) with sterilized lids and stored in a refrigerator (4 °C) until analyzed. Three batches of each YA were prepared independently.

### 2.3. Preparation of Milk Yogurt

Milk yogurt was also prepared by dissolving 0.10% (*w*/*w*) of pectin in a 150 mL aliquot of 2% milk (Highland Dairy, Springfield, MO, USA). Once the pectin was completely dissolved, the milk–pectin solution was blended with 3 quarts of 2% milk (Highland Dairy, Springfield, MO, USA) using a Vitamix Blender (Vitamix Drink Machine Plus, Cleveland, OH, USA) on the high-speed setting for two minutes. After blending, the mixture was poured into a Cuisinart stainless steel pot, 1.47% (*w*/*w*) of glucose and 8.16% (*w*/*w*) cane sugar were added, and the mixture was heated with constant stirring to 85 °C and maintained at that temperature for 10 min. Then, the same procedure as for rice flour YAs was followed. Three batches of yogurt made from milk were prepared independently.

### 2.4. Syneresis During Storage

Syneresis of YAs and milk yogurt was determined by centrifuging 30× *g* of yogurt at 2000 rpm at 5 °C [30] on day 1, 7, 14, 21, and 28. The amount of liquid released from the mixture was weighed and used to determine the syneresis % using the following equation:

$$Syneresis\;(\%)=\frac{weight\;of\;supernatant\;(g)}{weight\;of\;yogurt\;sample\;(g)}\times 100\%$$

### 2.5. Monitoring of Color During Storage

Color of YAs and milk yogurt samples was analyzed using a portable colorimeter (BC-10, Konica Minolta, Inc., Osaka, Japan) on day 1, 7, 14, 21, and 28. The colorimeter was calibrated using a white tile before analyzing samples. The YAs and milk yogurt were placed into 5-ounce glass dishes and placed on a flat surface, and then the colorimeter was covered a Ziploc bag. The bag was tightly held to ensure the lens was covered without any air pockets or wrinkles and then submerged into the YA. The color values L* (whiteness to blackness), a* (redness to greenness), b* (yellowness to blueness) were recorded.

### 2.6. Monitoring of TA During Storage

The titratable acidity (TA) of the YAs and milk yogurt samples was measured during storage on day 1, 7, 14, 21, and 28. The TA was measured using the standard titration method (AOAC 16.023) and expressed as % lactic acid. A mixture of 10 g of yogurt sample was measured in an Erlenmeyer flask, and 30 mL of deionized water was mixed until homogenous. The solution was mixed and titrated with 0.1 N NaOH using 0.1% phenolphthalein (Ricca Chemical, Inc., Arlington, TX, USA) as the indicator until the end point was reached, which was a faint pink color.

### 2.7. Monitoring of pH During Storage

The pH of the YAs and milk yogurt in glass jars was measured at day 1, 7, 14, 21, and 28 by using an Accumet AE150 pH Benchtop Meter (Fisher Scientific Instruments, Pittsburgh, PA, USA) that was calibrated with pH 4.00, 7.00, and 10.00 buffer solutions (Fisher Scientific Instruments, Pittsburgh, PA, USA).

### 2.8. Monitoring of Viscosity During Storage

Viscosity measurements on YAs and milk yogurt samples in glass jars were analyzed using a Brookfield DV-II viscometer (Brookfield Engineering Lab Inc., Stoughton, MA, USA) at day 1, 7, 14, 21, and 28. A T-C spindle was used with a helipath stand and rotated at 30 rpm with the temperature maintained at 5 ± 2 °C during testing. The results were obtained using Wingather 32 software (Brookfield Engineering Lab Inc., Stoughton, MA, USA). One hundred data points were averaged per sample [31].

### 2.9. Enumeration of Streptococcus thermophilus, Lactobacillus bulgaricus and Lactobacillus rhamnosus During Storage

To monitor the growth and survival of *Streptococcus thermophilus* (ST) bacteria in YAs, the procedures by Morris et al. [25] were employed. ST bacteria were analyzed on day 1, 7, 14, 21, and 28 for the YAs and milk yogurt. ST agar was prepared by adding the following reagents in 1 L of distilled water: 10 g of sucrose (Ultrapure, 99%, Alfa Aesar, Ward Hill, MA, USA), 5.0 g of Gibco^TM^ Bacto^TM^ Yeast Extract (Waltham, MA, USA), 10 g of Gibco^TM^ Bacto^TM^ Tryptone (Fisher Scientific, Waltham, MA, USA), and 2.0 g K_2_HPO_4_ (Fisher Scientific, Waltham, MA, USA). The mixture was dissolved on a stir plate at room temperature, then the pH was reduced to 6.8 ± 0.1 using 1N HCl solution. Then, 6 mL of 0.5% bromocresol purple (Fisher Scientific, St. Louis, MO, USA) and 12 g of agar (pure powder, Thermo Scientific Chemicals, Waltham, MA, USA) were added to the mixture. The mixture was then stirred and heated to boiling for 1 min. After heating, the solution was transferred to Duran glass bottles (Fisher Scientific, Waltham, MA, USA) and then autoclaved at 121 °C for 15 min. After autoclaving, the solution was placed into a 60 °C water bath and allowed to equilibrate to the water bath temperature. A mass of 10 g of YA or milk yogurt was diluted with 90 mL of sterilized peptone water. Then, it was serially diluted by pipetting 1 mL of solution into 9 mL of sterilized 0.1% peptone water in sterile centrifuge tubes. Then, 1 mL of each dilution was pipetted into a sterile petri dish, and then 12–15 mL of the melted ST agar was poured into the petri dish and mixed well. Petri dishes were then inverted and incubated aerobically at 37 °C for 48 h. ST bacteria were counted on the plates that displayed 30 to 300 colonies. To enumerate the colonies, they were counted using a Stuart Digital Colony Counter (Cole-Parmer, Vernon Hills, IL, USA). ST bacteria counts were expressed as log CFU/g.

To monitor the growth and survival of *Lactobacillus bulgaricus* (LB) bacteria in YAs and milk yogurt during storage [25], LB bacteria were analyzed on day 1, 7, 14, 21, and 28. Agar was prepared by dissolving 62 g of MRS agar (Oxoid, Basingstoke, Hampshire, UK) into 1 L of distilled water. The pH was then adjusted to 5.2 using 1.0 N HCl. The mixture was then stirred and heated to boiling for 1 min. After heating the mixture, the solution was transferred to Duran glass bottles and then autoclaved at 121 °C for 15 min. After autoclaving, the solution was placed into a 50 °C water bath and allowed to equilibrate to the water bath temperature. The same sample preparation and dilution process as for ST was followed. Petri dishes were then inverted and incubated anaerobically at 43 °C for 72 h. Bacteria counts for LB were obtained as mentioned previously for ST, using 10 g of YA.

To monitor the growth and survival of *Lactobacillus rhamnosus* (LGG) bacteria in YAs [25], YAs and milk yogurt samples were analyzed on day 1, 7, 14, 21, and 28 of storage. *Lactobacillus rhamnosus* agar was prepared by dissolving 62 g of MRS agar (Oxoid, Basingstoke, Hampshire, UK) into 1 L of distilled water. The pH was then adjusted to 6.5 using 1.0 N HCl. The mixture was then stirred and heated to boiling for 1 min. After the mixture was heated, the solution was transferred to Duran glass bottles and then autoclaved at 121 °C for 15 min. After autoclaving, the solution was placed into a 50 °C water bath and allowed to equilibrate to the water bath temperature. After the agar was autoclaved, the temperature was allowed to reach 50 °C. A solution of 50 ppm vanomycin solution was prepared and sterilized using a Whatman 0.22 µm disposable sterile syringe filter (General Electric) and a BD Leur-Lock Syringe (Avantor, Allentown, PA, USA). After sterilization, the vanomycin solution was added to the LGG agar. LGG was measured in YA samples initially after inoculating yogurt with bacteria and then every 45 min thereafter. The same sample preparation and dilution process as for ST was followed. Petri dishes were then incubated anaerobically at 37 °C for 48 h. LGG Bacteria counts were obtained as mentioned previously for ST, using 10 g of YA.

### 2.10. In Vitro Gastrointestinal Fluid (GIF) Tolerance

On day 1, 14, and 28, the YAs and milk yogurt samples were sampled to determine the probiotic survivability under simulated gastric and intestinal conditions. Simulated gastric juice (SGJ) and simulated intestinal juice (SIJ) was prepared using a modified version procedure by Ranadheera et al. [32]. SGJ was prepared by adding 3 g/L of Pepsin (1:10,000) (MP Biomedicals, Irvine, CA, USA) with 0.5% (*w*/*v*) NaCl, and the pH was adjusted to 3, following a standardized static in vitro method [33], with 1 M HCl. The simulated gastric juice was then filtered through a 0.22 µm sterile filter (Whatman, Cole-Parmer, Vernon Hills, IL, USA) attached to a Luer lock syringe (BD syringe). Tolerance of the probiotic bacteria to the SGJ was determined by placing 1 g of YA or milk yogurt into a 15 mL centrifuge tube, then adding 9 mL of SGJ and vortexing for 30 s. During simulated digestion, samples were incubated at 37 °C (100 rpm). One mL samples were taken at 0, 1, 90, and 180 min. After each time point during the in vitro digestion process, a 1 mL aliquot was removed from the appropriate tube and was immediately placed into 9 mL of 0.1% sterilized peptone water, vortexed, and then serially diluted. After the serial dilutions, the samples were plated using the LGG agar using the pour–plate method, as previously discussed.

Simulated intestinal juices (SIJ) were prepared by adding pancreatin (MP Biomedicals) 1 g/L, 0.5% NaCl (*w*/*v*) and 0.3% bile salts (Oxoid, Basingstoke, Hampshire, UK), and the pH was adjusted to 8.0 with 0.1 M NaOH. The simulated intestinal juice was then filtered through a 0.22 µm sterile filter (Whatman) attached to a Luer lock syringe (BD syringe). Tolerance of the probiotic bacteria to the SIJ was determined by placing 1 g of YA or milk yogurt into a 15 mL centrifuge, then adding 9 mL of SIJ and vortexing for 30 s. During simulated digestion, samples were incubated at 37 °C (100 rpm). One mL samples were taken at 0, 1, 120, and 240 min. After each time point during the in vitro digestion process, a 1 mL aliquot was removed from the appropriate tube and was immediately placed into 9 mL of 0.1% sterilized peptone water, vortexed, and then serially diluted. After making the serial dilutions, the samples were plated using the LGG agar using the pour–plate method, as previously discussed [32].

### 2.11. Yeast, Mold, and Coliforms Analysis During Storage

Yeast, mold, and coliforms microbiological analyses were conducted on the refrigerated YAs and milk yogurt samples on days 1, 7, 14, 21, and 28. Ten grams of sample from each treatment was removed from the sample jar, placed in 90 mL of 0.1% sterilized peptone water, and then serially diluted. For each treatment, serial dilutions were prepared, and samples were plated on both E.coli/Coliform and Rapid Yeast and Mold Petrifilms™ (3M Company, St. Paul, MN, USA). One mL of each dilution was pipetted onto the Petrifilm. A spreader was placed in the center of the film to distribute the sample evenly. Petrifilms were allowed to set for 5 min. The Petrifilms were incubated at 35° ± 1 °C for 48 h. and 25° ± 1 °C for 48 h, for Coliforms and yeast and molds, respectively. Colonies were counted using a Stuart Digital Colony Counter (Cole-Parmer, Vernon Hills, IL, USA). Yeast, mold, and coliform counts were express as log CFU/g.

### 2.12. Statistics

All analyses were carried out in triplicate. Three batches of each YA and MY were prepared for each treatment. Mean values and standard deviations were reported. Analysis of variance (ANOVA) was used to evaluate the differences between the treatment groups, followed by post hoc Tukey’s test using SAS (version 9.2). The statistical significance level was set to *p* ≤ 0.05.

## 3. Results and Discussion

### 3.1. Syneresis

Syneresis is considered to be a defect in yogurts and is defined as the serum discharged from the gel [34]. Table 1 shows the changes in the syneresis of yogurt alternatives (YAs) and MY (milk yogurt). During the storage study, commercial rice flour YAs (CMWRF-YA and CMBRF-YA) did not release any serum during the 28-day storage period. The FBRF-YA also did not release any serum up until day 21, and on day 28, the syneresis was 0.42 ± 0.04%. The syneresis level of FWRF-YA and MY increased with storage time; however, the serum released was significantly higher for MY (Table 1). Grasso et al. [35] observed lower water-holding capacity in milk-based yogurt than plant-based yogurt. The high syneresis values for the MY during storage may be attributed to the heating time of the MY mix. Typically, MY is heated at 80 °C for 30 min to denature the proteins [36]. Additionally, the bacteria used in the study were designed for plant-based products, which could affect the gel formation of the MY. Therefore, a weaker gel network may be the cause of the high syneresis for MY. The lower syneresis values of the YAs can be attributed to the high carbohydrate content in the rice flours [37]. Typically, starch increases the water-holding capacity. Starch is commonly used in yogurt production as a stabilizer and to prevent syneresis due to its swelling behavior when heated [38]. The results from this study displayed a lower syneresis level compared to a study conducted by Shi et al. [39] that reported between 19.61 and 43.37% syneresis for an almond yogurt formulated with pectin and xanthan gum. Syneresis was significantly higher and increased during storage for the yogurt made from milk when compared to the YAs. Yogurt made from milk has casein and whey. The protein network was rearranged as the pH decreased during storage, and this caused whey to be expelled from the gel matrix [40]. In a study by Román et al. [41], sauces made with rice flour or wheat flour at smaller particle sizes produced a weaker sauce with the highest syneresis for both flours. This phenomenon is relatable to the difference in syneresis for FWRF-YA and FBRF-YA (Table 1). Analysis of the FWRF and

FBRF showed that the mean particle sizes were 131.37 μm and 403 μm, respectively (Table 2). At the concentration of flour that was used, it could be deduced that the smaller particles for FWRF did not take up enough volume and could not form strong/cohesive structures. Larger particles could take up more space in the matrix, which may aid in increasing flow resistance, as noted by Roman et al. [41]. Aleman et al. [42] showed that rice flour with the largest particle size showed resistance to flow with the lowest G′ and G″ values. Arocas et al. [43] found that syneresis in sauces was highest for samples with the highest G′ and G″ values. Jiao et al. [44] found that yogurt made from unhomogenized walnut milk had a lower yield stress and water-holding capacity, resulting in a softer texture. These studies indicate that larger particle size is associated with soft gels and less chance for syneresis, while smaller particle size, like that found in FWRF, results in greater syneresis.

### 3.2. Color

The color values (L*, a* and b*) were measured during storage for 28 days. An increase in whiteness (L* value) was observed for FWRF-YA when comparing day 1 to day 28 (Table 3). There was no significant difference in L* values over time for the rest of the YAs and milk yogurt. There was a significant change in a* for CMWRF-YA and FWRF-YA during refrigerated storage when comparing day 1 to day 28. There was a significant increase in b* for FWRF-YA, CMBRF-YA, and MY when comparing day 1 to day 28, therefore making the product less blue and more yellow. Almusallam et al. [45] reported that changes in yogurt color values could be attributed to acidity and modifications in structure that can lead to the release of natural pigments that may surface and alter the color.

### 3.3. Titratable Acidity

**Figure 1:**
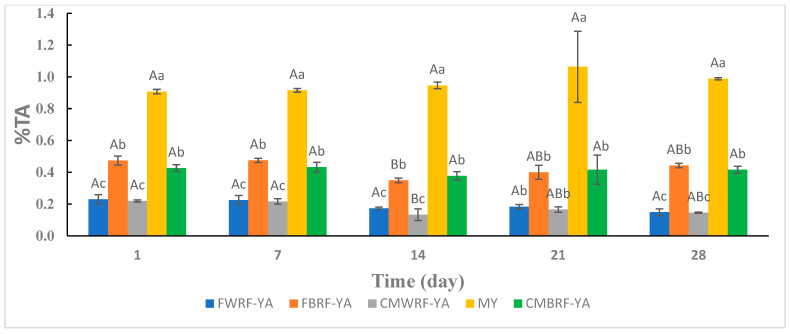
Titratable acidity of YAs and milk yogurt during refrigerated storage. ^a–c^ Means ± SD with the same letters between treatments at the same day are not significantly different. ^A,B^ Means ± SD with different letters within treatments at different days indicate significant difference. FWRF-YA = Frontière white rice flour yogurt alternative, CMWRF-YA = commercial white rice flour yogurt alternative, FBRF-YA = Frontière brown rice flour yogurt alternative, CMBRF-YA = commercial brown rice flour yogurt alternative, MY = yogurt made from milk.

The TA of yogurt alternatives containing rice flour was between 0.1 and 0.5% throughout storage (Figure 1). This is consistent with the values obtained by Plengsaengsri et al. [46], who found TA values between roughly 0.2 and 0.45% for rice-based yogurts during storage at 4 °C for 28 days. During storage, TA values in the present study were significantly higher for the milk-based yogurt (0.91–1.10%) compared with yogurt alternatives containing rice flour. Demïr et al. [47] produced a non-dairy oat yogurt that had %TA less than 0.20%, which is similar to what was reported in this study. Grasso et al. [35] performed a study comparing the physicochemical properties of a commercial dairy yogurt to plant-based (soy, coconut, cashew, almond, and hemp) yogurts. Their findings showed that the TA of milk-based yogurt was significantly higher than the plant-based yogurts. They suggested that perhaps lactose in dairy yogurt was a preferable substrate than plant-based yogurt carbohydrates for bacterial fermentation. Though all samples in the present study initially contained the same amount of added glucose and sucrose to boost fermentation, the milk-based yogurt substrate gave it the edge over rice flour substrates for enhanced production of acids. From day 1 to day 28 of storage, there was no significant difference in TA within treatments. This may be due to the bacteria strain that was used to ferment the samples [48]. The bacteria strain determines the degree of acidification (i.e., production of lactic acid) in yogurt products [49].

### 3.4. pH

**Figure 2:**
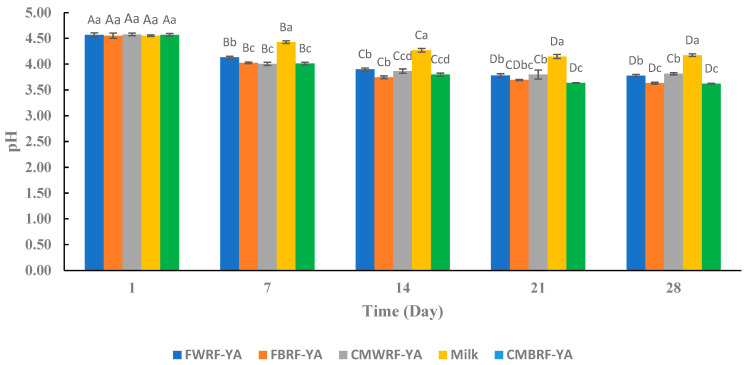
Changes in pH of YAs and milk yogurt during refrigerated storage. ^a–d^ Means ± SD with the same letters between treatments at the same day are not significantly different. ^A–D^ Means ± SD with different letters within treatments at different days indicate significant difference. FWRF-YA = Frontiére white rice flour yogurt alternative, CMWRF-YA = commercial white rice flour yogurt alternative, FBRF-YA = Frontiére brown rice flour yogurt alternative, CMBRF-YA = commercial brown rice flour yogurt alternative, MY = yogurt made from milk.

The pH of the YAs and MY post-fermentation was approximately 4.6. There was no significant difference in the pH between samples at day 1 of storage (Figure 2). After 28 days of storage, the pH was significantly lower in the brown rice flour yogurt alternatives in reference to the other samples. The pH of all the samples decreased significantly during storage due to the production of acidic compounds via the lactic acid bacteria [50] and/or protein hydrolysis, which generates amino acids and peptides [51]. The starter culture used to ferment all the samples consisted of a 1:1 of *Lactobacillus delbrueckii* ssp. *bulgaricus (LB)* and *Streptococcus thermophilus*. Together, these bacteria have a symbiotic relationship. Most strains of LB are known to have proteolytic activity [48]. The results showed that the YAs and yogurt produced from milk had a decrease in pH over the storage period of 28 days (Figure 2). There was a significant decrease in pH for all the samples from day 1 till day 14; from day 21 to day 28, there was no significant decrease in pH for all the treatments. The YAs ΔpH range was between 0.70 to 0.83, and the yogurt made from milk ΔpH was 0.31 from day 1 to day 14. The results from this study are similar to the results from Grasso et al. [35], who determined that during storage, some plant-based yogurts, such as those made from hemp and coconut, showed lower pH than milk-based yogurt.

### 3.5. Viable Cell Counts of ST, LB, and LGG Bacteria

**Figure 3:**
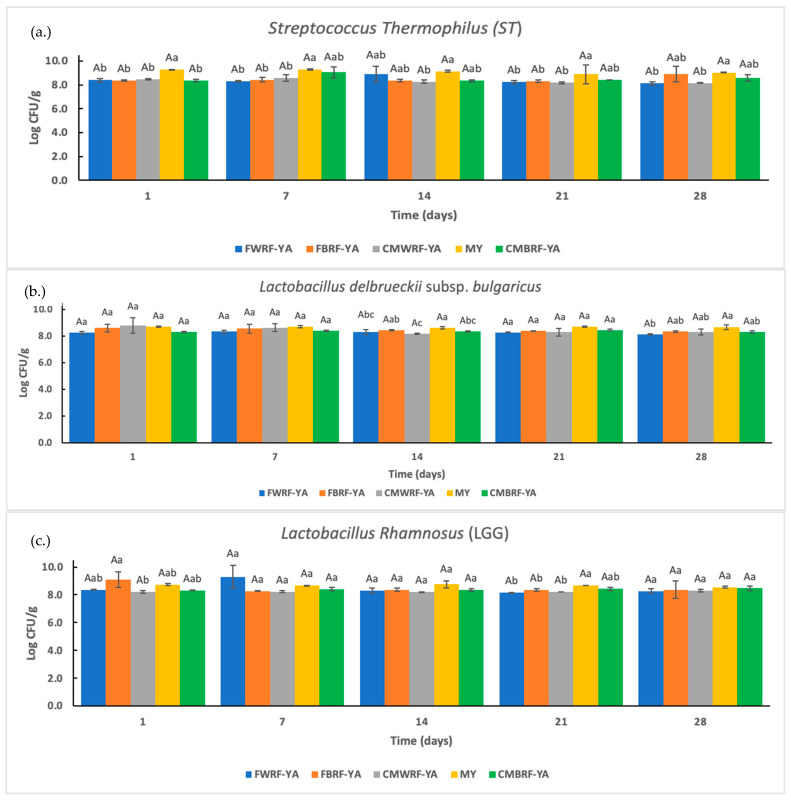
Bacteria viability during 28-day storage: (**a**) *Streptococcus thermophilus* (ST), (**b**) *Lactobacillus delbrueckii* subsp. *bulgaricus* (LB), and (**c**) *Lactobacillus rhamnosus* (LGG). ^a–c^ Means ± SD with the same letters between treatments at the same storage times are not significantly different. ^A^ Means ± SD with the same letters within treatments at different storage times are not significantly different. FWRF-YA = Frontière white rice flour yogurt alternative, CMWRF-YA = commercial white rice flour yogurt alternative, FBRF-YA = Frontière brown rice flour yogurt alternative, CMBRF-YA = commercial brown rice flour yogurt alternative, MY = yogurt made from milk.

The number of lactic acid bacteria (LAB) of YAs and yogurt made from milk during storage is shown in Figure 3. The number of ST, LB, and LGG bacteria remained above 8 log CFU/g for the entire storage study. There were no significant differences for storage time in all bacteria and very few differences between YA types within the same time. This may be due to the fact that the TA did not increase during storage. The bacteria growth rate is predicated on the lactic acid production [50]. Bacteria levels either increased or stayed the same during storage in a study on a fermented yogurt-like soy beverage [52]. Changes depended on the type of bacteria used. Counts of free LGG cells have been shown to decrease rapidly to non-detectable levels when stored at 4 °C for 150 days, whereas encapsulated cells maintained higher count levels [53]. Based on regulations set by the National Yogurt Association (NYA) [53], yogurt products must have at least 8 log cfu/g at the time of manufacture. Based on these guides, all of the samples would be able to receive the “live and active seal” [54].

#### Yeast Mold and Coliform in YAs and MY During Storage

Coliforms are indicator organisms to determine the quality of a food product and assess if Good Manufacturing Practices (GMP) were employed. Yeast, mold, and coliforms can affect the quality and physicochemical properties of food products. Acid produced from LAB can be utilized by yeast and mold and cause a decrease in acidity, which can lead to hydrolysis of proteins, breakdown of texture, and off-flavors [55]. Yeast, mold, and coliforms were not detected in yogurt made from milk. Red colonies were observed on the 3 M Petrifilm Rapid *E. coli*/Coliform plates for all the YAs on day 21 and 28 but were not counted as coliforms due to the absence of gas bubbles. Yeast was not present throughout the storage study for all the YAs. The presence of molds was not detected until day 21 to day 28 for the YAs. The mold count was significantly higher on day 28 (Table 4). The mold counts in the YAs ranged from 1.39 ± 0.27 to 2.42 ± 0.24 (Table 3). Yogurt is a perishable food product, and the permissible limits for coliform, mold, and yeast are <1 cfu/g, <1 cfu/g, and <10 cfu/g, respectively [56]. The presence of molds in the YAs may be an indicator of the YAs’ base mixtures not being heated for an adequate amount of time, improper sterilization of equipment and storage containers, or temperature abuse during storage [55]. It may be possible that cross contamination occurred somehow [57]. Optimization of pasteurization times and temperatures for unfermented rice flour mixtures may be needed. It might be possible to utilize different lactic acid bacteria to inhibit mold growth, as noted by [58], or chemical preservatives that inhibit mold growth might be needed.

### 3.6. Viscosity of YAs and MY During Storage

**Figure 4:**
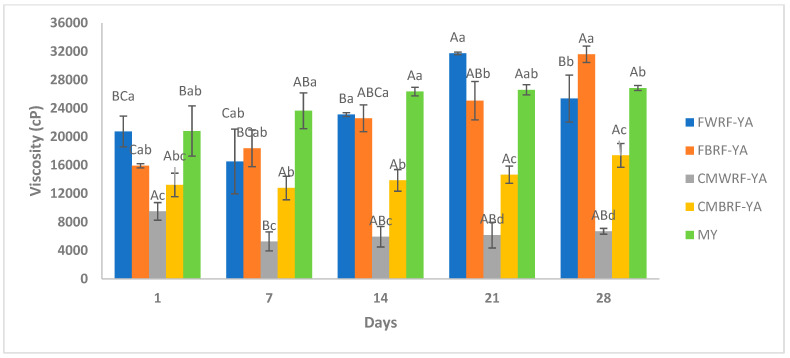
Apparent viscosity of YAs and MY during refrigerated storage. ^a–d^ Means ± SD with the same letters between treatments at the same storage times are not significantly different. ^A–C^ Means ± SD with different letters within treatments at different storage times indicate significant difference. FWRF-YA = Frontière white rice flour yogurt alternative, CMWRF-YA = commercial white rice flour yogurt alternative, FBRF-YA = Frontière brown rice flour yogurt alternative, CMBRF-YA = commercial brown rice flour yogurt alternate, YM = yogurt made from milk.

The results obtained from apparent viscosity showed that FWRF-YA, CMBRF-YA, and CMWRF-YA were not significantly different at the beginning (day 1) and the end (day 28) after the storage study (Figure 4). For the FBRF-YA, the apparent viscosity increased significantly from day 1 to day 28 of storage. The yogurt made from milk increased in viscosity from day 1 to day 14 during storage. Then, there was no significant difference in apparent viscosity for the reminder of the storage study. The FWRF-YA had a viscosity that was similar to the yogurt produced using milk. The YAs contained starch, which has the ability to gelatinize and increase viscosity [59]. FWRF-YA and FBRF-YA exhibited similar viscosities to yogurt produced from milk. Overall, the YAs displayed similar viscosities to Achanta et al. [31], who reported apparent viscosities made from yogurt produced from milk that ranged from 13,747–16,745 cP.

### 3.7. Gastrointestinal In Vitro Analysis of Lactobacillus rhamnosus (LGG) During Storage

MY had significantly higher LGG viable counts during one day of storage than other samples that were exposed to in vitro gastric conditions (Table 5). However, on day 14, MY was the only treatment that experienced a significant within-treatment decrease in LGG counts during exposure to in vitro gastric conditions. This decreasing trend shifted solely to CMBRF-YA at day 28, where significant decreases were observed at 90 and 180 min of incubation. The milk-based yogurt matrix was shown to initially have a buffering or protective effect for LGG during in vitro gastric simulations when compared with the rice-based yogurt alternatives. During storage, the MY sample suffered from syneresis, which may have affected its ability to protect LGG. Previous studies [28,29] indicated that starch can assist with water-holding capacity in plant-based yogurts to help protect the bacteria. Free LGG cells were shown to decrease from 11.4 CFU/g to 4.7 CFU/g after only 4 h exposure to gastric conditions [53]. Nevertheless, viable counts for all YA treatment/storage time/incubation time combinations remained high at >7.3 log CFU/g.

After consumption, conveyance of health benefits from probiotics to the host is facilitated by survival in an acidic gastric environment, travel to the small intestine, and colonization. *Lactobacillus* spp. are assisted in this regard due to their strong natural resistance to acid [60]. This intrinsic resistance is linked with the bacteria possessing a continual gradient between more-acidic extracellular and more-alkaline cytoplasmic pH values. Moreover, cellular functions cease, and cell death occurs, when an internal pH threshold value is reached [61]. The LGG probiotic strain has been shown to survive at very low pH (2.5) for 4 h [62]. The addition of glucose to rice flour yogurt alternatives in the current study may also have been advantageous in protecting LGG. Corcoran et al. [63] reported that the presence of metabolizable sugars (glucose) enhanced the survival of LGG and other probiotic *Lactobacillus* strains in acidic environments (simulated gastric juice, pH 2.0).

Between all treatments at days 1 and 14 of storage, the white rice-containing treatments (FWRF-YA and CMWRF-YA) and MY retained the highest LGG viability at longer in vitro intestinal incubation times (Table 6). At day 28 of storage, FWRF-YA and CMWRF-YA had significantly greater LGG retention than all other samples. There was a significant within-treatment decreasing trend in LGG counts for FBRF-YA, CMBRF-YA, and MY during 240 min incubation at each storage day. On the other hand, the white rice-containing treatments had significant within-treatment LGG losses only at day 14 for CMWRF-YA and day 1 and 14 for FWRF-YA. At time 0 of exposure to in vitro intestinal conditions, all treatment/storage sample combinations had around 8–9 log CFU/g of LGG. At the longest incubation (240 min) and storage (28 days) times, LGG counts dropped to 7.18 ± 0.50, 3.63 ± 0.26, 7.13 ± 0.48, 5.42 ± 0.26, and 3.09 ± 0.50 log CFU/g for FWRF-YA, FBRF-YA, CMWRF-YA, MY, and CMBRF-YA, respectively.

During exposure to in vitro intestinal conditions, LGG probiotic bacteria counts of FBRF-YA, MY, and CMBRF-YA dipped below the recommended minimum concentration (10^6^–10^7^ CFU/mL or g) in food products that would potentially allow the host to derive health benefits after consumption [32,64]. The sustained high viable LGG counts of FWRF-YA and CMWRF-YA are preferable for potentially providing consumers with probiotic-related health benefits. Oxgall (bile) works to emulsify and solubilize fats. When bacterial cell plasma membranes mostly composed of phospholipids are exposed to bile-containing environments, they can be lysed, leading to cell damage. Food matrices or food constituents can allow bacteria to survive better and resist the harmful effects of bile by binding bile acids [65]. Ashwar et al. [66] found that gelatinized rice starch samples displayed significantly greater bile binding ability than native rice starch. While all rice flour yogurt alternatives in the current study were gelatinized, the extent of gelatinization may have been higher for white rice flour samples. Incomplete gelatinization in FBRF and CMBRF could potentially account for the comparatively lower bile survival capacity of LGG. Gelatinization temperature for brown rice was reported to be higher compared to white rice [67]. FBRF and CBRF were found to have greater gelatinization temperatures than the corresponding white rice flours, which could be due to greater protein, which can inhibit starch swelling, and differences in the amylose levels of the flours [42].

## 4. Conclusions

There is an increasing trend to develop yogurt alternatives. In this study, YAs were derived from white and brown varieties of rice flour. A yogurt from milk was also produced. The YAs were able to maintain viable bacteria counts throughout the 28-day storage period; however, additional research needs to be conducted to determine how to minimize or eliminate the molds in the YAs. It may be necessary to add preservatives to limit mold growth and extend shelf-life. The titratable acidity was significantly lower when compared to the yogurt made from milk, but the syneresis for the YAs outperformed the yogurt made from milk. The YAs formulated with white rice flour also had significant survivability in the intestine, whereas YAs from brown rice flour and yogurt made from milk were below the recommended range of 10^6^ to 10^7^ CFU/g. Encapsulation of the bacteria before addition may result in slow release in the intestine and increase the counts to an acceptable range in these two samples. The pH and TA were lower in YAs when compared to yogurt made from milk. Lactic acid or lactose addition could possibly be utilized to increase the TA of the YAs.

## Figures and Tables

**Table 1:** Syneresis of yogurt alternatives and milk yogurt during refrigerated storage.

	Syneresis (%)				
Treatment	Storage Period (day)				
	1	7	14	21	28
FWRF-YA	0.07 ± 0.01 ^Db^	0.27 ± 0.02 ^Db^	5.13 ± 0.15 ^Cb^	10.60 ± 0.35 ^Bb^	16.79 ± 0.18 ^Ab^
FBRF-YA	0.00 ± 0.00 ^Bb^	0.00 ± 0.00 ^Bb^	0.00 ± 0.00 ^Bc^	0.00 ± 0.00 ^Bc^	0.42 ± 0.04 ^Ac^
CMWRF-YA	0.00 ± 0.00 ^Ab^	0.00 ± 0.00 ^Ab^	0.00 ± 0.00 ^Ac^	0.00 ± 0.00 ^Ac^	0.00 ± 0.00 ^Ad^
MY	35.79 ± 0.22 ^Aa^	37.11 ± 0.51 ^Aa^	37.54 ± 0.48 ^Aa^	36.07 ± 3.2 ^Aa^	38.24 ± 0.30 ^Aa^
CMBRF-YA	0.00 ± 0.00 ^Ab^	0.00 ± 0.00 ^Ab^	0.00 ± 0.00 ^Ac^	0.00 ± 0.00 ^Ac^	0.00 ± 0.00 ^Ad^

^a–d^ Means ± SD with the same letters between treatments at the same storage times are not significantly different. ^A–D^ Means ± SD with different letters within treatments at different storage times indicate significant difference. FWRF-YA = Frontière white rice flour yogurt alternative, CMWRF-YA = commercial white rice flour yogurt alternative, FBRF-YA = Frontière brown rice flour yogurt alternative, CMBRF-YA = commercial brown rice flour yogurt alternative, MY = yogurt made from milk.

**Table 2:** Particle size of rice flours.

	D (10) (µm)	D (50) (µm)	D (90) (µm)	MV (µm)
CMBRF	63.87 ± 10.87 ^A^	204.10 ± 58.77 ^AB^	371.87 ± 47.37 ^B^	230.10 ± 47.47 ^BC^
CMWRF	56.18 ± 1.05 ^A^	262.67 ± 76.89 ^AB^	417.40 ± 1.66 ^B^	295.33 ± 54.93 ^AB^
FBRF	47.51 ± 4.64 ^A^	304.70 ± 6.01 ^A^	1315 ± 224.34 ^A^	403.40 ± 53.58 ^A^
FWRF	20.96 ± 2.53 ^B^	132.53 ± 24.98 ^B^	235.30 ± 7.95 ^B^	131.37 ± 10.70 ^C^

Values are mean ± standard deviation, from triplicate determinations. ^A–C^ Means with different superscripts within a column differ significantly (*p* < 0.05). D (10) = the size of particle below which 10% of the sample lies, D (50) = the size in microns at which 50% of the sample is smaller and 50% is larger, D (90) = the size of particle below which 90% of the sample lies, MV = Mean Volume Diameter, FBRF = Frontière brown rice flour, FWRF = Frontière white rice flour, CMBRF = commercial brown rice flour, CMWRF = commercial white rice flour.

**Table 3:** Effect of storage on color parameter (L*, a* and b*) on YAs and milk yogurt during refrigerated storage.

Color Parameters	Treatment	Storage Period (day)				
		1	7	14	21	28
L*	FWRF-YA	73.03 ± 0.57 ^BCb^	72.57 ± 0.40 ^Cbc^	73.38 ± 0.24 ^ABCc^	74.00 ± 0.70 ^ABc^	74.29 ± 0.20 ^Ab^
	FBRF-YA	74.87 ± 1.44 ^Ab^	74.40 ± 1.28 ^Ab^	75.47 ± 0.08 ^Ab^	75.97 ± 0.59 ^Ab^	74.16 ± 0.05 ^Ab^
	CMWRF-YA	62.63 ± 0.15 ^ABd^	62.27 ± 0.68 ^Bd^	62.68 ± 0.19 ^ABe^	63.00 ± 0.30 ^ABe^	63.58 ± 0.20 ^Ad^
	MY	91.90 ± 0.72 ^Aa^	91.67 ± 0.90 ^Aa^	93.20 ± 0.17 ^Aa^	93.20 ± 0.36 ^Aa^	91.30 ± 1.04 ^Aa^
	CMBRF-YA	70.77 ± 0.72 ^Ac^	70.90 ± 0.46 ^Ac^	69.20 ± 0.10 ^Ad^	70.37 ± 1.21 ^Ad^	69.33 ± 0.06 ^Ac^
a*	FWRF-YA	−2.60 ± 0.00 ^Cb^	−2.50 ± 0.00 ^Cb^	−2.10 ± 0.06 ^Ab^	−2.23 ± 0.12 ^ABb^	−2.30 ± 0.00 ^Bc^
	FBRF-YA	−2.17 ± 0.06 ^Aab^	−2.10 ± 0.10 ^Aab^	−2.16 ± 0.06 ^Ab^	−2.13 ± 0.12 ^Ab^	−2.07 ± 0.12 ^Ab^
	CMWRF-YA	−2.07 ± 0.06 ^Aab^	−2.13 ± 0.12 ^ABab^	−2.17 ± 0.04 ^ABb^	−2.20 ± 0.10 ^ABb^	−2.33 ± 0.06 ^Bc^
	MY	−1.70 ± 0.46 ^Aa^	−1.70 ± 0.46 ^Aa^	−1.60 ± 0.10 ^Aa^	−1.70 ± 0.10 ^Aa^	−1.57 ± 0.06 ^Aa^
	CMBRF-YA	−2.47 ± 0.12 ^Ab^	−2.40 ± 0.17 ^Ab^	−2.16 ± 0.05 ^Ab^	−2.23 ± 0.15 ^Ab^	−2.20 ± 0.00 ^Abc^
b*	FWRF-YA	−1.73 ± 0.15 ^Bd^	−1.70 ± 0.10 ^Bd^	−0.80 ± 0.10 ^Ad^	−0.73 ± 0.15 ^Ac^	−0.86 ± 0.05 ^Ac^
	FBRF-YA	3.70 ± 0.66 ^Ab^	3.70 ± 0.66 ^Ab^	2.90 ± 0.00 ^Ab^	2.90 ± 0.10 ^Ab^	2.90 ± 0.10 ^Ab^
	CMWRF-YA	−2.50 ± 0.10 ^Ad^	−2.47 ± 0.06 ^Ad^	−2.20 ± 0.00 ^Ae^	−2.37 ± 0.31 ^Ad^	−2.37 ± 0.2 ^Ad^
	MY	7.53 ± 0.25 ^Ba^	7.47 ± 0.15 ^Ba^	8.07 ± 0.03 ^Aa^	8.23 ± 0.06 ^Aa^	8.03 ± 0.06 ^Aa^
	CMBRF-YA	2.27 ± 0.06 ^Bc^	2.23 ± 0.06 ^Bc^	2.70 ± 0.10 ^Ac^	2.67 ± 0.23 ^Ab^	2.82 ± 0.07 ^Ab^

^a–e^ Means ± SD with the same letters between treatments at the same storage day are not significantly different. ^A–C^ Means ± SD with different letters within treatments at different storage days indicate significant difference. FWRF-YA = Frontiére white rice flour yogurt alternative, CMWRF-YA = commercial white rice flour yogurt alternative, FBRF-YA = Frontiére brown rice flour yogurt alternative, CMBRF-YA = commercial brown rice flour yogurt alternative, MY = yogurt made from milk. The color values L*, a*, and b* represent the degree of (darkness/lightness), (redness/greenness), and (yellowness/blueness), respectively.

**Table 4:** Yeast, mold, and coliforms in YAs during refrigerated storage.

Treatment	Storage Period (day)				
	1	7	14	21	28
	Molds (log CFU/g)				
FWRF-YA	N.D.	N.D.	N.D.	1.69 ± 0.09 ^Ba^	2.33 ± 0.35 ^Aa^
FBRF-YA	N.D.	N.D.	N.D.	1.46 ± 0.15 ^Ba^	2.42 ± 0.24 ^Aa^
CMWRF-YA	N.D.	N.D.	N.D.	1.59 ± 0.11 ^Ba^	2.40 ± 0.46 ^Aa^
MY	N.D.	N.D.	N.D.	N.D.	N.D.
CMBRF-YA	N.D.	N.D.	N.D.	1.39 ± 0.27 ^Ba^	2.31 ± 0.15 ^Aa^
	Yeast (log CFU/g)				
FWRF-YA	N.D.	N.D.	N.D.	N.D.	N.D.
FBRF-YA	N.D.	N.D.	N.D.	N.D.	N.D.
CMWRF-YA	N.D.	N.D.	N.D.	N.D.	N.D.
MY	N.D.	N.D.	N.D.	N.D.	N.D.
CMBRF-YA	N.D.	N.D.	N.D.	N.D.	N.D.
	Coliforms (log CFU/g)				
FWRF-YA	N.D.	N.D.	N.D.	N.D.	N.D.
FBRF-YA	N.D.	N.D.	N.D.	N.D.	N.D.
CMWRF-YA	N.D.	N.D.	N.D.	N.D.	N.D.
MY	N.D.	N.D.	N.D.	N.D.	N.D.
CMBRF-YA	N.D.	N.D.	N.D.	N.D.	N.D.

^a^ Means ± SD with the same letters between treatments at the same storge times are not significantly different. ^A,B^ Means ± SD with different letters within treatments at different storage times indicate significant difference. FWRF-YA = Frontière white rice flour yogurt alternative, CMWRF-YA = commercial white rice flour yogurt alternative, FBRF-YA = Frontière white rice flour yogurt alternative, CMBRF-YA = commercial brown rice flour yogurt alternative, MY = yogurt made from milk, N.D. = not detected.

**Table 5:** Bacteria counts of LGG during in vitro gastric conditions during storage.

Storage Day	Time (min)	Viable Cell Counts (log CFU/g)				
		FWRF-YA	FBRF-YA	CMWRF-YA	MY	CMBRF-YA
1	0	8.37 ± 0.04 ^Aa^	8.77 ± 0.68 ^Aa^	8.23 ± 0.14 ^Aa^	8.71 ± 0.11 ^Aa^	8.32 ± 0.04 ^Aa^
1	1	8.20 ± 0.10 ^Ab^	8.17 ± 0.13 ^Ab^	8.15± 0.07 ^Ab^	8.63 ± 0.01 ^Aa^	8.30 ± 0.03 ^Ab^
1	90	8.18 ± 0.09 ^Ab^	8.17 ± 0.06 ^Ab^	8.16 ± 0.05 ^Ab^	8.61 ± 0.06 ^Aa^	8.12 ± 0.08 ^Ab^
1	180	8.20 ± 0.07 ^Ab^	8.19 ± 0.04 ^Ab^	8.12 ± 0.03 ^Ab^	8.57 ± 0.04 ^Aa^	8.01 ± 0.16 ^Ab^
14	0	8.21 ± 0.16 ^Ab^	8.33 ± 0.16 ^Ab^	8.17 ± 0.06 ^Ab^	9.19 ± 0.30 ^Aa^	8.33 ± 0.10 ^Ab^
14	1	8.16 ± 0.11 ^Ab^	8.30 ± 0.08 ^Aab^	8.14 ± 0.13 ^Ab^	8.61 ± 0.03 ^Ba^	8.38 ± 0.16 ^Aab^
14	90	8.07 ± 0.09 ^Ab^	7.78 ± 0.07 ^Ab^	7.92 ± 0.11 ^Ab^	8.49 ± 0.02 ^Ba^	8.08 ± 0.06 ^Ab^
14	180	8.09 ± 0.07 ^Aa^	7.30 ± 0.49 ^Aa^	7.96 ± 0.03 ^Aa^	8.52 ± 0.15 ^Ba^	7.85 ± 0.46 ^Aa^
28	0	8.18 ± 0.10 ^Aa^	8.01 ± 0.81 ^Aa^	8.25 ± 0.03 ^Aa^	8.52 ± 0.04 ^Aa^	8.37 ± 0.04 ^Aa^
28	1	8.23 ± 0.06 ^Aa^	8.47 ± 0.24 ^Aa^	8.17 ± 0.05 ^Aa^	8.54 ± 0.11 ^Aa^	8.41 ± 0.05 ^Aa^
28	90	8.02 ± 0.24 ^Aab^	7.89 ± 0.09 ^Aab^	7.79 ± 0.20 ^Ab^	8.49 ± 0.13 ^Aa^	8.15 ± 0.08 ^Bab^
28	180	7.53 ± 0.64 ^Aa^	7.68 ± 0.22 ^Aa^	7.90 ± 0.22 ^Aa^	8.57 ± 0.10 ^Aa^	7.91 ± 0.05 ^Ca^

^A–C^ Means ± SD with the same letters within treatments are not significantly different. ^a,b^ Means ± SD with different letters between treatments indicate significant difference. FWRF-YA = Frontière white rice flour yogurt alternative, CMWRF-YA = commercial white rice flour yogurt alternative, FBRF-YA = Frontière brown rice flour yogurt alternative, CMBRF-YA = commercial brown rice flour yogurt alternative, and MY = milk yogurt.

**Table 6:** Bacteria counts of LGG during in vitro intestinal conditions during storage.

Storage Day	Time (min)	Viable Cell Counts (log CFU/g)				
		FWRF-YA	FBRF-YA	CMWRF-YA	MY	CMBRF-YA
1	0	8.37 ± 0.04 ^Aa^	8.77 ± 0.68 ^Aa^	8.23 ± 0.14 ^Aa^	8.71 ± 0.11 ^Aa^	8.32 ± 0.04 ^Aa^
1	1	8.08 ± 0.03 ^ABa^	7.00 ± 0.03 ^Bb^	7.98 ± 0.20 ^Aa^	7.05 ± 0.17 ^Bb^	8.10 ± 0.05 ^Aa^
1	120	7.79 ± 0.07 ^ABa^	5.92 ± 0.11 ^BCb^	7.28 ± 0.52 ^Aa^	6.81 ± 0.04 ^Bab^	4.88 ± 0.14 ^Bc^
1	240	7.44 ± 0.36 ^Ba^	4.87 ± 0.04 ^Cb^	7.04 ± 0.50 ^Aa^	6.55 ± 0.21 ^Ba^	5.03 ± 0.07 ^Bb^
14	0	8.21 ± 0.16 ^Ab^	8.33 ± 0.16 ^Ab^	8.17 ± 0.06 ^Ab^	9.19 ± 0.30 ^Aa^	8.33 ± 0.10 ^Ab^
14	1	7.97 ± 0.18 ^Aab^	6.08 ± 0.06 ^Bc^	7.92 ± 0.04 ^ABab^	6.90 ± 0.05 ^Bbc^	8.48 ± 0.73 ^Aa^
14	120	7.10 ± 0.11 ^Ba^	3.89 ± 0.07 ^Cc^	7.43 ± 0.16 ^BCa^	6.28 ± 0.09 ^Cab^	4.23 ± 1.18 ^Bbc^
14	240	6.81 ± 0.30 ^Ba^	3.95 ± 0.08 ^Cb^	7.16 ± 0.26 ^Ca^	5.94 ± 0.23 ^Cab^	4.15 ± 1.06 ^Bb^
28	0	8.18 ± 0.10 ^Aa^	8.01 ± 0.81 ^Aa^	8.25 ± 0.03 ^Aa^	8.52 ± 0.04 ^Aa^	8.37 ± 0.04 ^Aa^
28	1	7.20 ± 0.79 ^Aa^	5.63 ± 0.28 ^ABab^	7.40 ± 0.28 ^Aa^	6.99 ± 0.20 ^Ba^	3.92 ± 0.88 ^Bb^
28	120	6.96 ± 0.90 ^Aa^	4.97 ± 1.87 ^ABa^	7.23 ± 0.40 ^Aa^	6.25 ± 0.38 ^BCa^	3.65 ± 0.33 ^Ba^
28	240	7.18 ± 0.50 ^Aa^	3.63 ± 0.26 ^Bc^	7.13 ± 0.48 ^Aa^	5.42 ± 0.26 ^Cb^	3.09 ± 0.50 ^Bc^

^A–C^ Means ± SD with the same letters within treatments are not significantly different. ^a–c^ Means ± SD with different letters between treatments indicate significant difference. FWRF-YA = Frontière white rice flour yogurt alternative, CMWRF-YA = commercial white rice flour yogurt alternative, FBRF-YA = Frontière white rice flour yogurt alternative, CMBRF-YA = commercial brown rice flour yogurt alternative, and MY = yogurt made from milk.

## Data Availability

The original contributions presented in this study are included in the article. Further inquiries can be directed to the corresponding author.
